# Small in Size, but Large in Action: microRNAs as Potential Modulators of PTEN in Breast and Lung Cancers

**DOI:** 10.3390/biom11020304

**Published:** 2021-02-18

**Authors:** Asal Jalal Abadi, Ali Zarrabi, Mohammad Hossein Gholami, Sepideh Mirzaei, Farid Hashemi, Amirhossein Zabolian, Maliheh Entezari, Kiavash Hushmandi, Milad Ashrafizadeh, Haroon Khan, Alan Prem Kumar

**Affiliations:** 1 Department of Genetics, Faculty of Advanced Science and Technology, Tehran Medical Sciences, Islamic Azad University, Tehran 193951495, Iran; Asal_jalali@yahoo.com (A.J.A.); entezarimali@yahoo.com (M.E.); 2 Sabanci University Nanotechnology Research and Application Center (SUNUM), Tuzla 34956, Istanbul, Turkey; alizarrabi@sabanciuniv.edu; 3 Faculty of Veterinary Medicine, Kazerun Branch, Islamic Azad University, Kazerun 7319866451, Iran; hoseingholami2020@yahoo.com; 4 Department of Biology, Faculty of Science, Islamic Azad University, Science and Research Branch, Tehran 193951495, Iran; sepidehmirzaei.smv@gmail.com; 5 Department of Comparative Biosciences, Faculty of Veterinary Medicine, University of Tehran, Tehran 1417466191, Iran; faridhashemi172@gmail.com; 6 Young Researchers and Elite Club, Tehran Medical Sciences, Islamic Azad University, Tehran 193951495, Iran; ah_zabolian@student.iautmu.ac.ir; 7 Department of Food Hygiene and Quality Control, Division of Epidemiology, Faculty of Veterinary Medicine, University of Tehran, Tehran 1417466191, Iran; houshmandi.kia7@ut.ac.ir; 8 Faculty of Engineering and Natural Sciences, Sabanci University, Orta Mahalle, Üniversite Caddesi No. 27, Orhanlı, Tuzla 34956, Istanbul, Turkey; 9 Department of Pharmacy, Abdul Wali Khan University, Mardan 23200, Pakistan; 10 NUS Centre for Cancer Research (N2CR), Yong Loo Lin School of Medicine, National University of Singapore, Singapore 119228, Singapore; 11 Cancer Science Institute of Singapore and Department of Pharmacology, Yong Loo Lin School of Medicine, National University of Singapore, Singapore 117599, Singapore

**Keywords:** microRNA, cancer therapy, PTEN, lung cancer, breast cancer, long non-coding RNA, circular RNA

## Abstract

MicroRNAs (miRNAs) are well-known regulators of biological mechanisms with a small size of 19–24 nucleotides and a single-stranded structure. miRNA dysregulation occurs in cancer progression. miRNAs can function as tumor-suppressing or tumor-promoting factors in cancer via regulating molecular pathways. Breast and lung cancers are two malignant thoracic tumors in which the abnormal expression of miRNAs plays a significant role in their development. Phosphatase and tensin homolog (PTEN) is a tumor-suppressor factor that is capable of suppressing the growth, viability, and metastasis of cancer cells via downregulating phosphatidylinositol 3-kinase (PI3K)/protein kinase B (Akt) signaling. PTEN downregulation occurs in lung and breast cancers to promote PI3K/Akt expression, leading to uncontrolled proliferation, metastasis, and their resistance to chemotherapy and radiotherapy. miRNAs as upstream mediators of PTEN can dually induce/inhibit PTEN signaling in affecting the malignant behavior of lung and breast cancer cells. Furthermore, long non-coding RNAs and circular RNAs can regulate the miRNA/PTEN axis in lung and breast cancer cells. It seems that anti-tumor compounds such as baicalein, propofol, and curcumin can induce PTEN upregulation by affecting miRNAs in suppressing breast and lung cancer progression. These topics are discussed in the current review with a focus on molecular pathways.

## 1. Introduction

Lung and breast cancers are malignant thoracic tumors. Lung cancer is a leading cause of death worldwide that has a 5-year survival rate as low as 18% [1]. In most cases of lung cancer (up to 80%), operation is not practical because of the delay in cancer diagnosis [2,3]. Consequently, a minor improvement has been achieved in survival rate. Annually, 220,000 patients with lung cancer are diagnosed in U.S.A where tobacco smoking is the major reason for its development [4,5,6]. Late diagnosis and metastasis into other vital organs of body such as the liver, bone, and nervous system are responsible for the poor prognosis of lung cancer patients [7,8,9]. Lung cancers are embedded to two major categories including small cell lung cancer (SCLC) and non-small cell lung cancer (NSCLC) in which NSCLC comprises most of lung cancer cases (up to 88%) [10,11]. Each of them has its subcategories. For instance, lung adenocarcinoma, lung squamous cell carcinoma, and large cell carcinoma are subcategories of NSCLC [10]. The normal structure of lung includes bronchiole and thin-walled alveoli surrounded by blood vessels. When lung cancers are developed, this normal structure is impaired by the penetration of tumor cells and stroma, providing an inflammatory response [7].

Similar to lung cancer, breast cancer remains a leading cause of death with high morbidity and mortality. According to estimates, one in eight British women are diagnosed with breast cancer [12,13,14]. The 5-year survival rate of breast cancer patients is dependent on stage, so that breast cancer patients in stage 1 or 2 have good 5-year survival rates of as much as 80%, but this number diminishes to 15% in stage 4 [12,15,16]. So, early diagnosis of breast cancer is of importance in its treatment and improving prognosis. Breast cancer is a heterogenous disease that can be divided into four categories based on the presence or absence of hormone receptors for estrogen, progesterone, and human epidermal growth factor receptor 2 (HER2) [15,16]. Lung and breast cancers are caused by multiple factors that have not been understood completely [17,18]. However, attempts have been conducted in improving knowledge toward genetic factors responsible for the development and progression of these thoracic cancers. MicroRNAs (miRNAs), as non-coding and short RNA molecules, are considered as potential diagnostic, therapeutic, and prognostic factors for breast and lung cancers [19,20]. There are two major types including tumor-suppressor and tumor-promoting miRNAs whose roles in the development of breast and lung cancers have been elucidated [21,22,23,24]. In the case of lung cancer, miRNAs have demonstrated capability to affect proliferation and metastasis. In this way, numerous factors are affected by miRNAs. For instance, miRNA-195 and miRNA-497 can disrupt lung cancer progression and colony formation via upregulating transforming growth factor-beta (TGF-β) [25]. There are also miRNAs that facilitate lung cancer malignancy. miRNA-143-3p stimulates N6-methyladenosine in elevating the brain metastasis of lung cancer cells [26]. Bone metastasis of lung cancer cells can be inhibited by miRNA-192-5p via the negative regulation of TRIM44 [27]. When the growth and migration of lung cancer cells enhance, they can induce chemoresistance. miRNA-27b suppresses epithelial-to-mesenchymal transition (EMT) via Snail downregulation to reverse chemoresistance [28].

A similar story is observed in breast cancer cells. Both tumor-suppressor and tumor-promoting miRNAs have been recognized in breast cancer. Tumor-promoting ones such as miRNA-532-5p elevate breast cancer proliferation via ras-related and estrogen-regulated growth inhibitor (RERG) downregulation [29]. In contrast, tumor-suppressor miRNAs such as miRNA-539 prevent breast cancer proliferation via specificity protein 1 (SP1) inhibition [30]. It is noteworthy that miRNA-7 is capable of suppressing the activity and expression of drug transporters including multidrug resistance protein 1 (MRP1) in inducing chemosensitivity [31]. Studies demonstrate that miRNAs are key players in breast cancer [32,33], and investigating their expression is of interest in providing therapeutics.

The role of miRNAs in breast and lung cancers is due to capability in regulating molecular pathways and cellular events. Dysregulation in miRNA expression is correlated with cancer development [34,35,36]. In the present review, our aim is to reveal the role of miRNAs in the progression/inhibition of lung and breast cancer cells, with a focus on their relationship with phosphatase and tensin homolog (PTEN). This review is based on newly recorded articles and providing a new insight toward signaling networks involved in lung and breast cancers in which miRNAs and PTEN are key players.

## 2. MicroRNAs in Oncology

The function of miRNAs in regulating gene expression is mediated by attachment to 3′-untranslated region (3′-UTR) of messenger RNA (mRNA) to inhibit gene expression [37,38,39,40]. The complexity of miRNA function is due to the capability of miRNAs to affect more than one mRNA and the presence of several miRNA binding sites at one 3′-UTR. Numerous miRNAs have been recognized to date with multiple functions [41]. The first discovery of miRNAs occurred in Caenorhabditis elegans, and significant research revealed conserved miRNAs in other species, including human with different actions [42]. In addition to development, miRNAs participate in the regulation of precise and accurate cellular events including apoptosis [43], autophagy [44], differentiation [45], migration [46], angiogenesis [47], and so on.

As an explosion has been observed in research about miRNAs, not it is obvious that miRNAs are therapeutic targets in cancer therapy. As normal and cellular events are regulated by miRNAs, and complicated signaling networks comprising upstream and down-stream mediators are involved, miRNA expression disturbance is correlated with cancer development [48,49,50]. Such pathways and roles have been examined in different cancers to shed some light on the relationship between miRNA expression and cancer emergence. It has been reported that one miRNA can affect the expression of another one. For instance, miRNA-145 enhances the expression of miRNA-133b via promoter methylation caused by c-Myc and DNMT3A [51]. Tumor-promoting miRNAs enhances cancer growth and provide ignorance of cancer cells toward apoptosis [52]. Serum levels of such miRNAs such as miRNA-1290 and miRNA-1246 can be considered as diagnostic factors [53]. In contrast, there are miRNAs with an inhibitory impact on cancer growth. miRNA-181a significantly increases cisplatin sensitivity in cervical cancer cells via apoptosis induction [54]. miRNA-200c enhances breast cancer sensitivity to trastuzumab via stemness inhibition [55]. It can be highlighted that the proliferation and metastasis of cancer cells are modulated by miRNAs [56,57]. Those miRNAs that promote cancer malignancy are involved in chemoresistance [58], while tumor-suppressor miRNAs induce chemosensitivity [59]. In fact, miRNAs regulate cellular events, and dysregulation in their expression leads to cancer emergence. In this way, all aspects of cancer cells such as growth, invasion, and their response to therapy are affected by miRNAs. Notably, molecular pathways such as nuclear factor erythroid 2–related factor 2 (Nrf2) [60], Wnt [61], Signal Transducer And Activator Of Transcription 3 (STAT3) [62], and Zinc Finger E-Box Binding Homeobox (ZEB) [63] are a few of the down-stream targets of miRNAs in cancer. Furthermore, there are molecular pathways that are capable of functioning as upstream mediators and regulating the expression of miRNAs such as circular RNAs (circRNAs) [64], and long non-coding RNAs (lncRNAs) [65]. Revealing such interactions can pave the road into effective cancer therapy that is the aim of this review article.

## 3. PTEN in Oncology

### 3.1. Signaling

**Figure 1:**
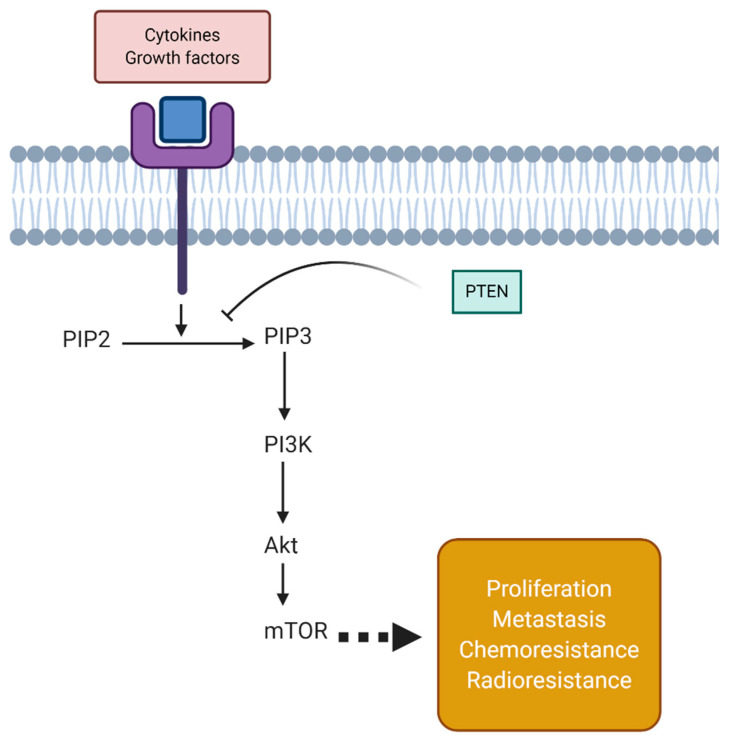
An overview of phosphatase and tensin homolog (PTEN) signaling. The cytokines and growth factors can induce the transformation of phosphatidylinositol-4,5-bisphosphate (PIP2) to phosphatidylinositol3,4,5-triphosphate (PIP3) by binding to a related receptor. Then, PI3K stimulates protein kinase B (Akt)/mammalian target of rapamycin (mTOR) signaling to induce the expression of genes involved in cancer progression, chemoresistance, and radioresistance. PTEN as a tumor-suppressing factor prevents PIP2 transformation to PIP3, restricting cancer malignancy.

PTEN is a tumor suppressor factor with nucleus and cytoplasmic localization with various expressions in healthy and cancerous cells [66,67]. In order to provide a better understanding of PTEN signaling, first, the phosphatidylinositol 3-kinase (PI3K)/protein kinase B (Akt)/mammalian target of rapamycin (mTOR) axis should be described. At the first step of this axis, p110 induces the conversion of phosphatidylinositol-4,5-bisphosphate (PIP2) into phosphatidylinositol3,4,5-triphosphate (PIP3) through phosphorylation of the 3′-hydroxyl group [68,69,70]. PIP3 is an inducer of PI3K and leads to the recruitment of Akt to membrane via binding their pleckstrin homology (PH) domains to PIP3 [71,72]. This interaction with PIP3 prevents the autoinhibition of Akt via phosphorylation at T308 by PDK1 and phosphorylation at S473 via mTOR [73,74,75]. This axis is further involved in promoting cancer cell proliferation, metastasis, and chemoresistance [9,76,77,78,79,80,81]. The cytoplasmic function of PTEN comprises of preventing PIP3 generation and inhibiting phosphorylation [82]. This action of PTEN suppresses the phosphorylation of p53 and activation of p21, resulting in preventing cell senescence. In addition to cytoplasmic functions, PTEN possesses nuclear functions including regulating genome stability and DNA repair (Figure 1) [83]. PTEN mutation in mice leads to genomic and chromosomal instability, revealing the nuclear function of this tumor suppressor factor that is independent of the PI3K/Akt/mTOR axis [84].

### 3.2. Role in Cancer

As a negative regulator of PI3K/Akt/mTOR signaling, and having a tumor-suppressor role, the downregulation of PTEN expression can induce the malignant behavior of cancer cells [85]. A newly published experiment has shown that PTEN loss is correlated with resistance to CDK4/6 inhibitors via Akt stimulation [86]. It appears that PTEN loss in hair follicle stem cells leads to the development of squamous cell carcinoma, showing the tumor-suppressor role of PTEN [87]. When a decrease occurs in PTEN expression, the proliferation and viability of cancer cells undergoes an increase. This is due to the inhibition of glycolysis by PTEN as a factor involved in the promoted growth of cancer cells [88]. The interesting point is that anti-tumor compounds including cryptotanshinone suppress cancer proliferation and induce apoptosis via PTEN upregulation and the subsequent inhibition of PI3K/Akt/mTOR and nuclear factor-kappaB (NF-ĸB) pathways [89]. Dichloroacetate can suppress chemoresistance in cancer cells via the downregulation of miRNA-543, upregulation of PTEN, and inhibition of the PI3K/Akt axis [90]. EMT is associated with the metastasis of cancer cells [81,91]. PTEN inhibits EMT via Abi downregulation, which is of importance in disrupting breast cancer progression [92]. Studies are in line with the fact that both the proliferation and migration of cancer cells are negatively affected by PTEN [93,94]. As PTEN possesses anti-tumor activity, its downregulation is associated with undesirable prognosis [95]. In breast cancer, PTEN hypermethylation is associated with the risk of breast cancer development and can be used as a reliable biomarker in this case [96]. One clinical study demonstrates an enhanced incidence of PTEN hypermethylation in breast cancer patients [97]. The same phenomenon occurs for lung cancer, and PTEN hypermethylation is observed upon the progression and chemoresistance development of lung cancer cells [98].

Importantly, non-coding RNAs are potential upstream mediators of PTEN in different cancers. For instance, lncRNA Linc00702 inhibits cancer progression via enhancing PTEN expression and suppressing the PI3K/Akt axis [99]. In turn, lncRNA LINC00470 accelerates cancer proliferation through providing PTEN degradation [100]. Similar to lncRNAs, circular RNAs (circRNAs) are able to regulate PTEN expression in affecting cancer progression [101]. Notably, numerous studies have shed light on the relationship between miRNAs and PTEN. Apoptosis, autophagy, and proliferation of cancer cells are affected by miRNAs in different cancers [102,103]. As miRNAs are well-known regulators in cancer cells, understanding their impact on PTEN expression can be beneficial in providing novel therapeutics. In the next sections, a mechanistic discussion of PTEN regulation by miRNAs in breast and lung cancers is provided.

## 4. MicroRNA and PTEN Relationship

### 4.1. MicroRNAs and PTEN Inhibition

#### 4.1.1. Breast Cancer

As PTEN has an inhibitory impact on the progression of breast cancer cells, its downregulation can occur by tumor-promoting miRNAs. miRNA-106b and miRNA-93 are potential factors in enhancing cancer growth and invasion via PTEN downregulation. This axis leads to activation of the PI3K/Akt pathway, which promotes cancer malignancy [104]. As PI3K/Akt participates in enhancing cancer growth, the way is paved to inducing chemoresistance. This statement can be confirmed by the effect of miRNA-2020-5p on PTEN expression in breast cancer cells. The miRNA-202 family has a dual role in cancer, exerting both tumor-promoting and tumor-suppressor roles [105]. As miRNA-202 demonstrates upregulation in human endometrium and adipose tissue-derived stem cells, it can be concluded that this miRNA family is involved in cell cycle regulation [106]. miRNA-202-5p undergoes upregulation in drug-resistant breast cancer cells, while PTEN shows a decrease in expression. By an increase in miRNA-202-5p, the proliferation and drug resistance of breast cancer cells enhance, while apoptosis is inhibited. These malignant behaviors are mediated by PTEN downregulation via miRNA-202-5p and the subsequent induction of PI3K/Akt signaling [107]. Notably, clinical studies have also confirmed a relationship between miRNAs and PTEN. In this way, miRNA-144 has been shown to be upregulated in 22% of breast cancer cases, and PTEN has a low expression in 78% of cases. There is a negative relationship between PTEN and miRNA-144 in migratory breast cancer cells [108]. The downregulation of PTEN by microRNAs is mediated by binding to 3′-UTR. Introducing PTEN that lacks 3′-UTR for miRNA promotes its expression and suppresses breast cancer progression [109].

Exosomes are vesicle-shaped structures with a diameter of 50–150 nm that can transport miRNAs as cargo. Various exosomal miRNAs have been identified in breast cancer such as exosomal miRNA-455-5p, -1255a, and -148a that can be used as therapeutic and diagnostic factors [110,111]. Exosomal miRNAs are capable of regulating PTEN expression in ensuring breast cancer malignancy. It seems that exosomal miRNA-9 and miRNA-155 possess high expression in metastatic breast cancer cells. This increase in the aggressive behavior of breast cancer cells is mediated via downregulating PTEN [112]. It is worth mentioning that PTEN-regulating miRNAs can be considered as potential diagnostic factors in breast cancer. Serum levels of miRNA-214 as a regulator of PTEN can provide distinction between malignant and benign tumors, and healthy cells. Furthermore, the expression level of miRNA-214 undergoes downregulation after operation [113]. A same story occurs for miRNA-21, so that the expression of this miRNA is high in advanced stages and is associated with lymph node metastasis. Following miRNA-21 upregulation, the expression of PTEN demonstrates a decrease of as much as 80% [114]. The increased expression of miRNA-425-5p, as a negative regulator of PTEN, is observed in breast cancer that is associated with unfavorable prognosis [115].

Cancer stem cells (CSCs), also known as cancer-initiating cells (CICs), possess self-renewal and multipotent differentiation potential that comprise a small proportion of tumor cells [116,117]. In breast cancer, CD44^+^/CD24^−^ are considered as surface markers of breast cancer stem cells (BCSCs) [118,119]. Carcinogenesis, migration, and chemoresistance are mediated by BCSCs [120]. Previously, it was demonstrated that miRNA-222 and miRNA-221 are negative regulators of PTEN in breast cancer progression. It appears that the aforementioned miRNAs possess regulatory impacts on BCSCs. By downregulating PTEN, miRNA-222 and miRNA-221 induce Akt phosphorylation to promote the growth and viability of breast cancer cells. miRNA-222 and miRNA-221 overexpression result in the enrichment of surface markers of CD44^+^/CD24^−^ in BCSCs [121]. This study demonstrates that in addition to cancer cells [122], CSCs are also affected by miRNA and PTEN interaction. miRNA-10b functions as a double-edged sword in cancer. It exerts both tumor-promoting and tumor-suppressing roles in cancer [123,124,125]. In breast cancer, miRNA-10b possesses a tumor-promoting role by affecting CSCs. miRNA-10b maintains the self-renewal capacity of BCSCs by PTEN downregulation and paving the way for Akt activation. The prolonged activation of Akt leads to an increase in the self-renewal capacity and expression of cancer stem cell markers that are in favor of breast cancer malignancy [126].

The identification of miRNAs targeting PTEN is of interest in providing novel therapeutics. For instance, miRNA-182-5p diminishes PTEN expression in increasing breast cancer survival and invasion. Silencing miRNA-182-5p is correlated with an increase in PTEN expression and suppressing breast cancer malignancy [127]. The interesting point is that both the proliferation and metastasis of breast cancer cells are affected by the relationship between miRNA and PTEN. The overexpression of miRNA-29b results in apoptosis inhibition and cancer metastasis via PTEN downregulation [128]. It is noteworthy that miRNAs can diminish the impact of environmental factors in breast cancer development. Phthalates (PAEs) are endocrine-disrupting compounds, and their role in breast cancer progression and initiation has been confirmed [129,130]. Exposing breast cancer cells to butyl benzyl phthalate is correlated with an increase in proliferation, transition from the G1 to S phase in the cell cycle, cyclin D1, the proliferation of cell nuclear antigen (PCNA), and a decrease in p21 expression. The investigation of molecular pathways demonstrates that butyl benzyl phthalate can bind to 3′-UTR of PTEN in reducing its expression, which is of importance for activating Akt and decreasing p21 expression [131]. These signaling networks provide breast cancer progression during exposure to environmental factors.

FOXO3a is an important member of the FOXO family with anti-tumor activity. In order to exert its inhibitory effect on cancer progression, FOXO3a should be stabilized in the nucleus. FOXO3a inhibition leads to breast cancer carcinogenesis [132]. FOXO3a overexpression is a desirable factor for prognosis, and its downregulation occurs in drug-resistant breast cancer cells [133]. Increasing evidence demonstrates that FOXO3a is a down-stream target of Akt [134,135,136]. As a tumor-promoting factor, miRNA-21 downregulates the expression of PTEN to induce Akt activation. Consequently, Akt stimulates the translocation of FOXO3a from the nucleus to the cytoplasm to prevent its anti-tumor action. Following FOXO3a downregulation, expressions of miRNA-34b and miRNA-34c undergo downregulation to increase CDK4 and CDK6 expression in favoring breast cancer progression [137]. Therefore, miRNAs are potential regulators of PTEN in breast cancer cells affecting proliferation, metastasis, and immune evasion [138]. These studies are in agreement with the fact that PTEN and its upstream and downstream mediators are in stringent surveillance of miRNAs affecting breast cancer progression and development (Table 1).

#### 4.1.2. Lung Cancer

Both miRNAs and PTEN can be considered as diagnostic and prognostic factors in lung cancer. For instance, miRNA-494 overexpression is associated with the poor prognosis, pathological tumor node metastasis (TNM), and lymph node metastasis of lung cancer cells. Furthermore, PTEN is associated with grade of differentiation [143]. Although this study has not evaluated miRNA and PTEN relationship in lung cancer, it shows that their expression is a determining factor for malignant behavior of lung cancer cells. Therefore, it is of significant importance to reveal miRNA and PTEN associations in lung cancer. The metastasis and growth of lung cancer cells mainly depend on the miRNA/PTEN axis. It has been reported that miRNA-106a binds to 3′-UTR of PTEN to reduce its expression, leading to lung cancer progression [144]. Decreasing the expression of such miRNAs causes the anti-apoptotic and pro-metastatic impacts to disappear by PTEN upregulation [145]. The aim of tumor-promoting miRNAs in PTEN inhibition is to activate PI3K/Akt signaling in increasing lung cancer progression [146]. Clinical studies have also confirmed miRNA and PTEN interaction in determining prognosis. It seems that miRNA-93-5p upregulation is correlated with poor prognosis via PTEN downregulation [147]. It is noteworthy that miRNAs can synergistically regulate PTEN in lung cancer progression. miRNA-21 and miRNA-155 synergistically induce PTEN downregulation in enhancing lung cancer progression [148].

It was mentioned that miRNAs affect PTEN in triggering PI3K/Akt signaling. It appears that downstream targets of Akt play a significant role in lung cancer progression. S-phase kinase-associated protein 2 (Skp2) is a member of F-box family, and its overexpression in lung cancer cells mediates their resistance to cisplatin chemotherapy [149]. As a tumor-promoting factor, miRNA-1297 reduces PTEN expression to activate Akt signaling, leading to Skp2 expression and the malignant behavior of lung cancer cells [150]. It is worth mentioning that miRNAs can regulate the proliferation of cancer cells by targeting glycolysis. Hexokinase 2 (HK2) and pyruvate kinase isozyme M2 (PKM2) are two important enzymes in glycolysis. HK2 is involved in the first step of glycolysis and is a rate-limiting enzyme [151], while PKM2 participates in the last step of glycolysis [152]. By PTEN downregulation, miRNA-214 induces PI3K/Akt signaling, leading to HK2 and PKM2 upregulations, and paving the way for the progression of lung cancer cells [153]. Therefore, the growth, viability and invasion of lung cancer cells are mainly regulated by the miRNA/PTEN axis [154].

One of the interesting points is the relationship between PTEN and the immune system in cancer [155]. It has been reported that PTEN loss is associated with the activation of programmed death-ligand 1 (PD-L1), mediating the immune evasion of cancer cells [156]. miRNA-142-5p can reduce the cytotoxicity of CD4+ cells against lung cancer via PTEN inhibition. PI3K/Akt and PD-L1 activations occur following miNRA-142-5p upregulation in lung cancer [157]. Therefore, the miRNA/PTEN axis not only affects the proliferation and invasion of lung cancer but also regulates immune response. It is noteworthy that the response of lung cancer cells to radiotherapy can also be regulated by the miRNA/PTEN axis. In this way, miRNA-181a downregulates PTEN expression to promote the progression and malignancy of lung cancer cells, resulting in their resistance to radiotherapy [158]. Downregulating miRNA-21 and miRNA-95 expressions promote PTEN expression to suppress PI3K/Akt signaling, resulting in the radiosensitivity of lung cancer cells [159]. It has been reported that lung cancer cells can secrete extracellular vesicles for transferring miRNAs. The miRNA-23a transferring leads to PTEN downregulation in lung cancer cells exposed to radiation, leading to angiogenesis [160].

STAT3 and PTEN demonstrate interactions in cancer cells. IL-8 can reduce PTEN expression via phosphorylation to stimulate STAT3 signaling, resulting in enhanced cancer progression [161]. Furthermore, STAT3 can function as an upstream mediator of PTEN by activating lncRNA cancer susceptibility candidate 9 (CASC9) to diminish PTEN expression, resulting in bladder cancer progression [162]. On the other hand, miRNAs such as miRNA-551b-3p can induce STAT3 signaling in enhancing the growth and metastasis of cancer cells [163]. Future studies can evaluate how miRNAs affect PTEN and STAT3 interaction in lung cancer. Taking everything into account, experiments demonstrate that miRNAs are versatile molecules in lung cancer by regulation PTEN signaling and affecting proliferation, invasion, and therapy response (Table 2) [164,165,166,167,168,169,170].

### 4.2. MicroRNAs and PTEN Induction

#### 4.2.1. Breast Cancer

**Figure 2:**
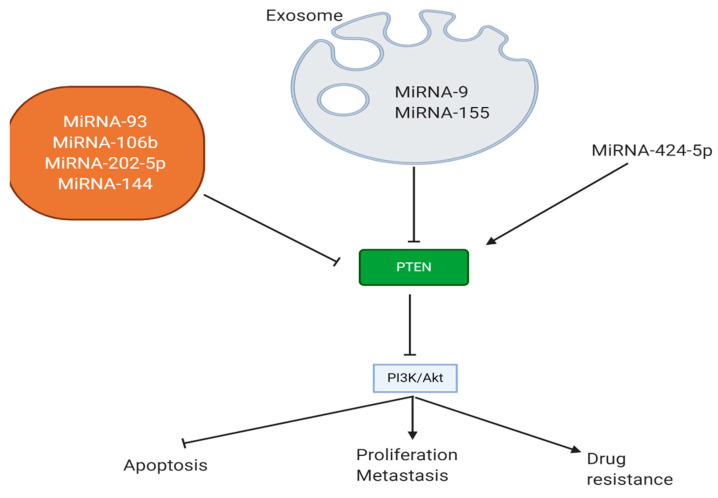
miRNAs suppressing/inducing PTEN expression in breast cancer, and affecting the progression, viability, and response of cancer cells to therapy.

miRNAs capable of promoting PTEN expression are considered as a tumor-suppressing factor in breast cancer. To date, most of the studies have focused on revealing the role of tumor-promoting miRNAs in breast cancer progression. However, a newly published experiment has investigated the efficacy of miRNA-424-5p in breast cancer therapy. This tumor-suppressing miRNA diminishes colony formation, cell viability, and the proliferation of breast cancer cells, and it induces apoptosis. In this way, miRNA-424-5p promotes PTEN expression to downregulate PI3K/Akt/mTOR signaling, resulting in breast cancer suppression (Figure 2) [179]. However, we still have a long way in revealing the role of miRNAs in suppressing PTEN expression.

#### 4.2.2. Lung Cancer

**Figure 3:**
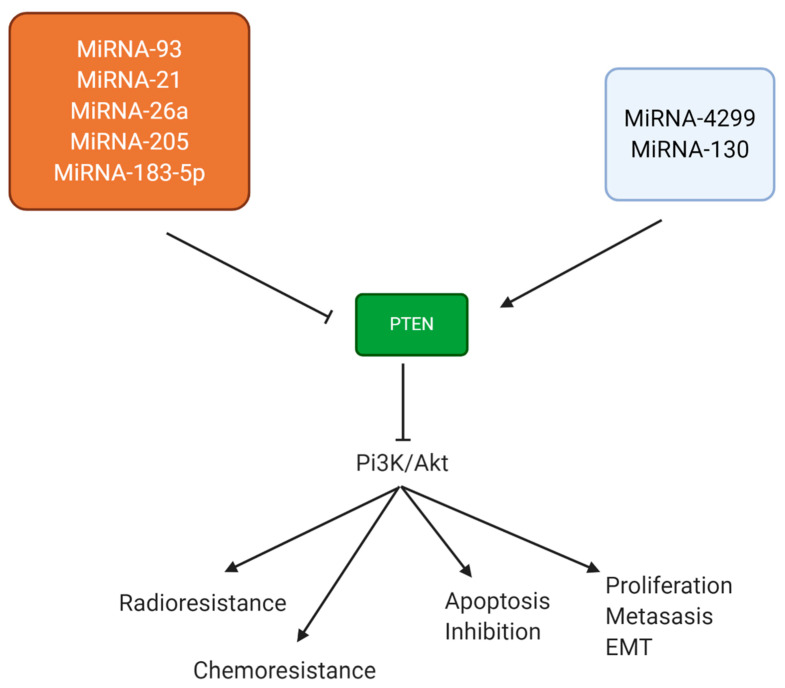
Radioresistance, chemoresistance, apoptosis, and metastasis of lung cancer cells are mainly affected by the miRNA/PTEN axis.

In lung cancer cells, PTEN induction is a negative factor for proliferation and metastasis. miRNA-4299 is a new emerging miRNA in lung cancer that is capable of promoting PTEN expression. The downregulation of miRNA-4299 occurs in lung cancer cells, and it is associated with TNM stage, histological grade, and lymph node metastasis. Enhancing miRNA-4299 expression is associated with good prognosis and can suppress the proliferation and migration of lung cancer cells via PTEN upregulation and the subsequent inhibition of PI3K/Akt signaling [180]. miRNA-130 is another important miRNA in lung cancer, but its exact role has not been completely understood. It has been reported that miRNA-130 can function as a tumor-promoting factor via inducing enhancer of zeste homolog 2 (EZH2) expression [181], while another study focuses on the tumor-suppressing role of miRNA-130 in lung cancer, showing that it can induce apoptosis in lung cancer cells and impair their proliferation via PTEN upregulation [182]. Similar to breast cancer, most studies have focused on revealing the role of tumor-promoting miRNAs in PTEN inhibition, and more studies are needed in the identification of miRNAs inducing PTEN signaling in lung cancer suppression (Figure 3).

### 4.3. MicroRNAs, PTEN, and Chemotherapy

#### 4.3.1. Breast Cancer

One of the preferred strategies in breast cancer therapy is chemotherapy. However, research is not always in favor, and increasing evidence demonstrates the capability of breast cancer cells to develop chemoresistance [183,184,185,186,187,188]. miRNAs have demonstrated a potential contribution in breast cancer chemoresistance. miRNA-30c triggers chemoresistance in breast cancer cells via histone deacetylase 9 (HDAC9) upregulation [189]. On the other hand, tumor-suppressor miRNAs such as miRNA-200c-3p are downregulated by lncRNA X-inactive specific transcript (XIST) in mediating chemoresistance [190]. miRNA-222 is suggested to be involved in inducing chemoresistance in breast cancer cells via affecting PTEN. The overexpression of miRNA-222 is associated with PTEN downregulation, paving the way for Akt upregulation and subsequent inhibition of p27^kip1^. This axis provides Adriamycin resistance in breast cancer cells [191]. This signaling network is more complicated when it is found that Akt can affect more down-stream targets. Akt is capable of activating NF-ĸB via phosphorylating IĸB kinase (IKK) [192,193,194,195]. NF-ĸB can induce cyclooxygenase-2 (COX-2) expression, which is an obvious finding in different malignancies [196,197,198,199]. As tumor-promoting factors, miRNA-222 and miRNA-221 reduce PTEN expression to elevate stem-cell properties and the proliferation of breast cancer cells. PTEN inhibition results in Akt activation, upregulating NF-kB and inducing COX-2, which are of importance for enhancing breast cancer malignancy [140].

In addition to COX-2, FOXO family members can be affected by Akt. FOXO1, FOXO3, and FOXO4 are members of the FOXO transcription family. Akt is capable of phosphorylating FOXO1 to provide the translocation of FOXO1 at the route of nucleus to cytoplasm, where it is degraded by the ubiquitin–proteasome pathway [200,201]. Upon PTEN activation, the expression of Akt is inhibited, and FOXO1 enters the nucleus, where it induces cell cycle arrest and apoptosis [202]. Such interactions are important for the drug resistance of breast cancer cells. It has been reported that miRNA-222 as a tumor-promoting factor triggers Akt phosphorylation via PTEN downregulation. This leads to a decrease in FOXO1 expression and level in the nucleus, which is of importance for enhancing chemoresistance in breast cancer cells [203]. Several experiments were discussed examining the role of miRNA-222 in triggering chemoresistance in breast cancer cells. Now, it is completely obvious that miRNA-222 is tumor-promoting in breast cancer, and it can promote proliferation and chemoresistance [204]. It can be concluded that the expression of tumor-promoting miRNAs such as miRNA-19 undergo upregulation in drug-resistant breast cancer cells, while a decrease occurs in PTEN expression [205].

One of the interesting points of drug resistance is the relationship between factors regulating the proliferation and metastasis of cancer cells. It seems that when tumor-suppressing factors regulating cancer proliferation are downregulated, the way for the upregulation of metastatic factors is paved. Such association has been investigated in the drug resistance of breast cancer cells. It has been reported that EMT induction can trigger the chemoresistance of cancer cells [206,207,208]. In breast cancer cells, miRNA-93 demonstrates an increase in expression that mediates the downregulation of PTEN, resulting in EMT induction and subsequent obtaining of drug resistance [209]. Although just one study has evaluated the EMT and PTEN relationship and their regulation by miRNAs in the drug resistance of breast cancer cells, we have still a long way in the identification of more miRNAs. For instance, p53 is a apoptosis-related factor that can function as an upstream mediator of PTEN in cancer therapy [210]. The combination of anti-miRNA-222/221 with cisplatin induces p53 expression to stimulate PTEN, resulting in increased efficacy in the eradication of triple-negative breast cancer cells [211].

The tumor-promoting miRNAs reduce the expression of PTEN in inducing chemoresistance. It seems that PTEN downregulation is associated with the resistance of cancer cells to chemotherapy-mediated apoptosis. The underlying molecular pathways involved in this kind of chemoresistance have been revealed. miRNA-222 can promote the resistance of breast cancer cells to Adriamycin chemotherapy via PTEN downregulation and the subsequent induction of Akt, leading to p27 inhibition and decreasing apoptotic cell death [191]. A same strategy is followed by miRNA-202-5p in inducing doxorubicin resistance, so that this miRNA significantly diminishes PTEN expression to stimulate PI3K/Akt signaling, resulting in apoptosis inhibition and providing chemoresistance [107]. Hence, the application of anti-tumor compounds capable of inducing apoptosis can be considered as a promising strategy in chemosensitivity. Overall, miRNAs are divided into two major groups, inducers and inhibitors of PTEN, that affect the response of breast cancer cells to chemotherapy (Table 3) [212].

#### 4.3.2. Lung Cancer

Due to the malignant behavior of lung cancer cells in terms of proliferation and metastasis, they can obtain resistance to chemotherapy [213]. Increasing evidence demonstrates the role of miRNAs in lung cancer cells acquiring chemoresistance [214,215]. Furthermore, PTEN downregulation occurs in drug-resistant lung cancer cells [216,217]. In this section, the association of miRNA with PTEN signaling in regulating the response of lung cancer cells to chemotherapy is discussed.

Cisplatin resistance is an increasing challenge in the treatment of lung cancer [218,219]. Autophagy as a “self-digestion” mechanism is suggested to be involved in the chemoresistance of lung cancer cells [220,221,222,223,224]. miRNA-181 as a tumor-suppressing factor inhibits autophagy via light chain-3 (LC3) and autophagy-related gene 5 (ATG5) downregulation. This is mediated via PTEN upregulation and the subsequent inhibition of PI3K/Akt signaling [225]. However, autophagy can also sensitize lung cancer cells to chemotherapy [226,227]. This dual role of autophagy and its association with the miRNA/PTEN axis can be considered in further experiments. Apoptosis induction and impairing proliferation are two major pathways followed by miRNAs inducing PTEN in providing the cisplatin sensitivity of lung cancer cells [228].

In enhancing the chemosensitivity of lung cancer cells, silencing the expression of tumor-promoting miRNAs is of importance. It has been reported that miRNA-23a downregulation paves the way for erlotinib sensitivity via PTEN upregulation. Upon PTEN activation, PI3K/Akt signaling inhibition occurs, impairing lung cancer progression [229]. miRNA-21 is one of the most important miRNAs in lung cancer, and its association with chemoresistance has been investigated in several studies. Increasing evidence demonstrates miRNA-21 involvement in enhancing cancer proliferation and metastasis via inducing molecular pathways such as Akt and matrix metalloproteinases (MMPs). Anti-tumor compounds such as sinomenine reduce miRNA-21 expression in disrupting lung cancer progression [230,231]. By PTEN downregulation, miRNA-21 promotes the expression of Akt and extracellular-signal regulated kinase (ERK) pathways, leading to the gefitinib resistance of lung cancer cells [232]. Upon hypoxic conditions, exosomal transfer of miRNA-21 occurs that subsequently mediates the resistance of lung cancer cells to cisplatin chemotherapy [233]. So, the most important pathway that miRNA-21 follows in inducing the chemoresistance of lung cancer cells is PTEN downregulation and the subsequent induction of PI3K/Akt signaling [234,235].

miRNA-1269b is a new emerging miRNA in cancer with an oncogene role, and it is capable of increasing cancer growth and invasion via Akt phosphorylation [236]. Cisplatin-resistant lung cancer cells demonstrate an increase in the expression of miRNA-1269b. An examination of molecular pathways shows that miRNA-1269b enhances cancer proliferation in vitro and in vivo and is correlated with chemoresistance. For this purpose, miRNA-1269b reduces PTEN expression to induce PI3K/Akt signaling [237]. The PTEN downregulation by miRNAs occurs via binding to 3′-UTR [238]. Interestingly, the proliferation and metastasis of cancer cells are in close relationship with each other and can trigger chemoresistance [239,240,241]. It has been reported that TGF-β can induce EMT in mediating chemoresistance [242,243,244,245]. miRNA-134/487b/655 stimulates TGF-β-mediated EMT in lung cancer cells. Then, the downregulation of membrane-associated guanylate kinase, WW, and PDZ domain-containing protein 2 (MAGI2) occurs, leading to PTEN loss and the gefitinib resistance of lung cancer cells [246]. Overall, studies are in agreement with the role of the miRNA/PTEN axis in regulating the response of lung cancer cells to chemotherapy (Table 4) [247,248].

### 4.4. Regulation of microRNA/PTEN Axis

#### 4.4.1. Breast Cancer

As miRNAs can regulate PTEN expression in breast cancer, and this is of importance in cancer proliferation and invasion as well as response of cancer cells to chemotherapy, experiments have focused on revealing the role of upstream mediators regulating the miRNA/PTEN axis in breast cancer cells.

lncRNAs are an important part of ncRNAs with a length more than 200 nucleotides capable of regulating miRNAs in breast cancer [250]. Furthermore, lncRNAs regulate PTEN in affecting the proliferation and metastasis of breast cancer cells [251,252]. LncRNA PTENP1 is a tumor-promoting factor in breast cancer that not only affects breast cancer progression but also influences drug sensitivity. LncRNA PTENP1 reduces PTEN expression via miRNA-20a sponging to upregulate PI3K/Akt signaling, resulting in breast cancer proliferation, metastasis, and adriamycin resistance [253]. In reducing the expression of miRNAs, lncRNAs can function as competing endogenous RNA (ceRNA). Although previous study demonstrated a tumor-promoting role of PTENP1 in breast cancer, another study reveals the tumor-suppressing role of this important lncRNA. LncRNA PTENP1 upregulates PTEN expression by miRNA-19b inhibition via sponging. Then, Akt downregulation and p53 upregulation occur to restrict the proliferation and metastasis of breast cancer cells [171]. It seems that lncRNA PTENP1 functions as a double-edged sword in breast cancer, and its exact role is not certain. However, it can effectively regulate breast cancer progression via affecting the miRNA/PTEN axis [254]. By the identification of tumor-promoting lncRNAs, they can be targeted in further studies for suppressing breast cancer progression. For instance, lncRNA GAS5 triggers tamoxifen resistance via miRNA-222 sponging and the subsequent inhibition of PTEN. Silencing GAS5 impairs breast cancer progression and enhances their sensitivity via activating the miRNA-222/PTEN axis [255]. To date, studies have focused on the recognition of tumor-promoting lncRNAs such as HOXC13-AS and ZFAS1, and further studies can identify tumor-suppressing lncRNAs regulating the miRNA/PTEN axis. It is obvious that (A) tumor-suppressing miRNAs are downregulated by lncRNAs in breast cancer progression, (B) PTEN downregulation occurs, (C) the way is paved for inducing factors involved in breast cancer malignancy such as PI3K/Akt, (D) breast cancer cells promote their proliferation and invasion, and (E) finally, they can obtain resistance to chemotherapy [36,256].

It is worth mentioning that in addition to lncRNAs, circular RNAs (circRNAs) can regulate the miRNA/PTEN axis in breast cancer cells. To date, two studies have evaluated the regulatory impact of circRNAs on the miRNA/PTEN axis in breast cancer cells that are included here. CircSLC8A1 is an inhibitor of cancer progression by regulating miRNAs and enhancing PTEN in bladder cancer therapy [257]. This circRNA exerts anti-tumor activity in breast cancer cells. By sponging miRNA-671, circSLC8A1 activates PTEN expression to inhibit PI3K/Akt signaling, limiting breast cancer progression [258]. Similar to lncRNAs, circRNAs reduce the expression of target miRNAs via sponging. This provides the condition for the activation of PTEN signaling and subsequent inhibition in the proliferation and invasion of breast cancer cells [259].

In addition to lncRNAs and circRNAs, other molecular pathways can function as upstream mediators of the miRNA/PTEN axis in breast cancer cells. Tumor necrosis factor-related apoptosis-inducing ligand (TRAIL) is a member of the tumor necrosis family (TNF) family that is capable of inducing apoptosis in cancer cells and is a promising target in cancer therapy [260]. However, it has been reported that cancer cells can obtain resistance to TRAIL-mediated apoptosis [261]. It seems that TRAIL resistance can trigger EMT in breast cancer cells to promote their metastasis and malignancy. In TRAIL-resistant cancer cells, miRNA-221 undergoes upregulation that subsequently reduces the expression of PTEN [262]. This study is also in line with previous experiments showing that the proliferation and invasion of breast cancer cells are in close relationship and the miRNA/PTEN axis plays a significant role. One of the hallmarks of cancer is the tumor microenvironment. Cancer-associated fibroblasts (CAFs) are the main stromal components of cancer cells with a potential role in cancer progression [190,263,264,265]. In breast cancer cells, CAFs can secrete exosomes containing miRNA-22 to bind to PTEN, reducing its expression and mediating tamoxifen resistance [266]. Therefore, upstream mediators of the miRNA/PTEN axis should be considered in breast cancer cells for developing novel therapeutics [267,268].

#### 4.4.2. Lung Cancer

One of the well-known tumor-suppressing lncRNAs in lung cancer is growth arrest-specific transcript 5 (GAS5). Enhancing the expression of lncRNA GAS5 effectively disrupts the proliferation and migration of lung cancer cells via miRNA-205 downregulation and enhancing PTEN expression [269]. It is noteworthy that lncRNA GAS5 can regulate the response of lung cancer cells to chemotherapy via modulating the miRNA/PTEN axis. For this purpose, lncRNA GAS5 reduces miRNA-21 expression to induce PTEN signaling [270]. Increasing evidence demonstrates that lung cancer cells, due to their aggressiveness and uncontrolled proliferation and metastasis, can obtain resistance to radiotherapy. The potential of GAS5 in providing radiosensitivity has been evaluated. By reducing miRNA-21 expression, lncRNA GAS5 induces apoptosis in lung cancer cells exposed to radiotherapy. It seems that PTEN upregulation and the subsequent inhibition of Akt signaling play a significant role in this case [271]. In contrast, tumor-promoting lncRNAs promote lung cancer progression via regulating the miRNA/PTEN axis. LncRNA LEF1-AS1 undergoes upregulation in lung cancer patients. This lncRNA promotes miRNA-221 expression to inhibit PTEN signaling, leading to proliferation inhibition and apoptosis induction in lung cancer cells [272]. Therefore, the identification of lncRNAs regulating the miRNA/PTEN axis can be of importance in developing novel therapeutics in lung cancer therapy [252,266]. One of the hallmarks of cancer cells is their alteration in metabolism. In order to meet their high need for energy, they utilize glycolysis [273]. CircRNAs can regulate the miRNA/PTEN axis in targeting the glucose uptake and metabolism of lung cancer cells. CircLARP4 reduces miRNA-135b expression to induce PTEN signaling. Then, it inhibits the Akt/HIF-1a axis to induce apoptosis in lung cancer cells and impair glycolysis [274].

NF-kB signaling is a regulator of biological mechanisms, mainly the immune system, and it can induce inflammation in promoting cancer progression [275,276,277]. Targeting and suppressing NF-kB signaling can significantly reduce lung cancer viability and proliferation [278,279,280,281,282]. It has been reported that NF-kB promotes miRNA-548as-3p expression to induce PTEN downregulation. Then, the way is paved for PI3K/Akt induction to promote the proliferation of lung cancer cells [283]. However, we are still at the beginning point, and more studies will reveal upstream mediators of the miRNA/PTEN axis [284].

### 4.5. MicroRNA/PTEN Axis: A Target of Anti-Tumor Compounds

#### 4.5.1. Breast Cancer

One of the interesting points of the miRNA/PTEN axis is its targeting by anti-tumor compounds. Most of the anti-tumor compounds evaluated in breast cancer therapy are phytochemicals targeting the miRNA/PTEN axis. In this section, we provide a mechanistic discussion of the role of naturally occurring compounds with anti-tumor activity in regulating the miRNA/PTEN axis.

Curcumin is a plant derived-natural compound derived from Curcuma longa with anti-tumor activity against breast cancer cells capable of inducing apoptosis and suppressing metastasis [285,286,287,288,289,290]. Curcumin is an important regulator of cell cycle in cancer. It seems that curcumin administration can mainly result in cell cycle arrest of cancer cells in the G2/M phase [291,292]. Curcumin can induce apoptosis in cancer cells via activating caspase cascade and upregulating caspase-3 expression [293]. It has been reported that curcumin can regulate miRNA expression in cancer therapy [294,295,296]. Upon curcumin administration, the expression level of tumor-promoting miRNAs including miRNA-21 and miRNA-27a undergoes downregulation, while an increase occurs in the expression of tumor-suppressing miRNAs such as miRNA-22 and miRNA-145 [297]. As PI3K/Akt activation is a common finding in cancer, it has been reported that curcumin administration downregulates PI3K/Akt expression via inducing PTEN signaling [298]. Curcumin can suppress the progression of chemoresistant-cancer cells via enhancing PTEN expression [299]. Curcumin administration suppresses breast cancer proliferation and stimulates cell cycle arrest at the G1/S phase. Mechanistically, curcumin downregulates the expression of miRNA-19a and miRNA-19b to induce PTEN signaling, leading to Akt downregulation and providing conditions for breast cancer therapy [300]. It seems that curcumin exerts its anti-tumor activity in a time- and dose-dependent manner. Exposing breast cancer cells to curcumin is correlated with miRNA-21 downregulation, the subsequent induction of PTEN, and the upregulation of caspase-3/9 in impairing breast cancer progression [301]. The important downstream target that is affected by the miRNA/PTEN axis is PI3K/Akt signaling. For this purpose, thidiazuron activates the miRNA-202-5p/PTEN axis to suppress PI3K/Akt signaling, leading to breast cancer inhibition [302].

Cantharidin (CTD) is a well-known compound in traditional Chinese medicine that can suppress cancer proliferation via triggering DNA damage [303]. It can induce apoptosis in cancer cells and reverse chemoresistance [304,305]. In breast cancer cells, CTD inhibits cancer progression in a time-dependent manner. CTD decreases the expression of miRNA-160b-93 as a tumor-promoting factor to enhance the expression of its downstream target PTEN, resulting in breast cancer inhibition [306]. Matrine is also an alkaloid derived from Sophora flavescens with capability in regulating the expression of miRNAs in cancer therapy [307]. It is noteworthy that it has been reported that matrine can regulate the miRNA/PTEN axis in colorectal cancer therapy [231], which is the same strategy that is followed in breast cancer therapy. In a time- and dose-dependent manner, matrine reduces breast cancer proliferation and triggers cell cycle arrest at the G1/S phase. Via miRNA-21 inhibition, matrine promotes PTEN expression to induce Akt dephosphorylation, leading to an accumulation of Bad, p21 and p27 in breast cancer therapy [308].

**Figure 4:**
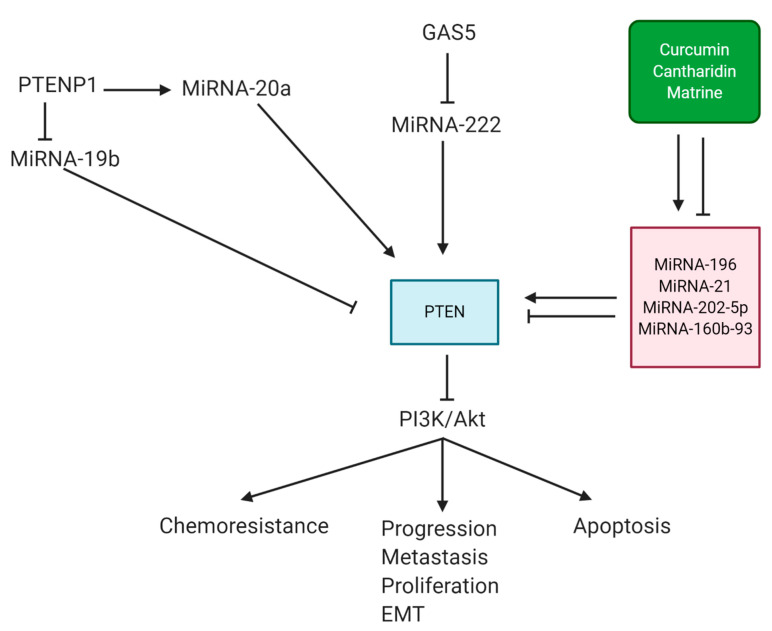
Anti-tumor compounds such as curcumin, cantharidin, and matrin target miRNAs in affecting the PTEN/PI3K/Akt axis in breast cancer therapy. Long non-coding RNAs (LncRNAs) such as PTENP1 and growth arrest-specific transcript 5 (GAS5) function as the main upstream mediators of the miRNA/PTEN axis in breast cancer.

Based on the published experiments, the following points can be concluded:

- Phytochemicals can be considered as epigenetic drugs in regulating miRNA expression,
- By targeting miRNAs, natural compounds can modulate PTEN expression,
- PTEN upregulation can impair PI3K/Akt signaling as the important pathway required for cancer progression,
- Apoptosis induction and proliferation inhibition are the major outcomes of using phytochemicals targeting the miRNA/PTEN axis in breast cancer therapy (Figure 4) [309,310,311,312,313].

#### 4.5.2. Lung Cancer

**Figure 5:**
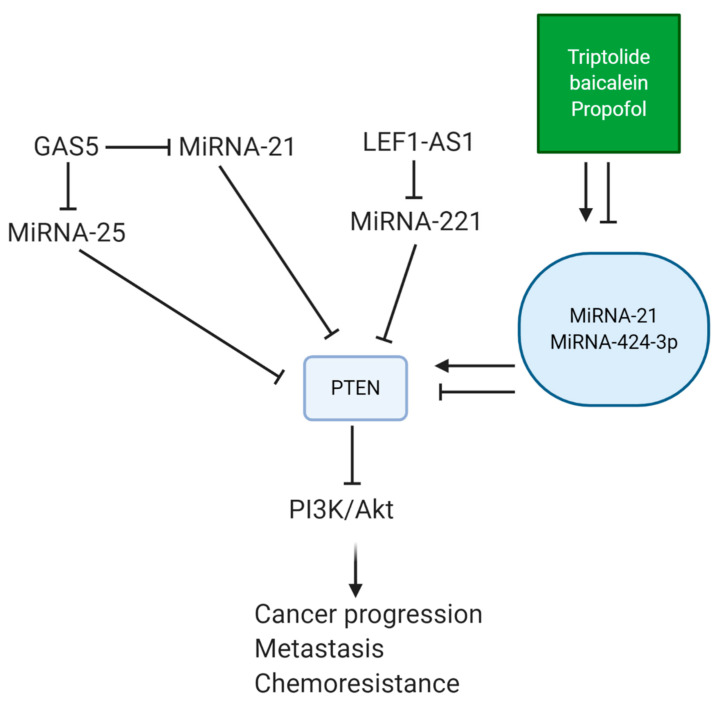
Similar to breast cancer, anti-tumor compounds and lncRNAs can regulate the miRNA/PTEN axis in affecting the progression of lung cancer cells.

Similar to breast cancer, anti-tumor compounds can regulate the miRNA/PTEN axis in affecting lung cancer progression. In this way, the expression of tumor-suppressing miRNAs undergoes downregulation, while an increase occurs in the expression of tumor-promoting miRNAs. Triptolide is a potent anti-tumor agent that has demonstrated inhibitory effect on cancer progression via targeting molecular pathways. Triptolide administration impairs the metastasis of lung cancer cells via EMT inhibition and reducing the expression levels of matrix metalloproteinase-9 (MMP9) [314]. This plant-derived natural compound induces apoptosis in lung cancer cells via miRNA-204-5p upregulation and the subsequent inhibition of Akt signaling [315]. In enhancing PTEN expression, triptolide promotes miRNA-21 expression to induce apoptosis in lung cancer cells, impairing their proliferation and viability [316]. The regulation of the miRNA/PTEN axis by anti-tumor compounds is of importance in enhancing the chemosensitivity of lung cancer cells. Baicalein administration is correlated with the cisplatin sensitivity of lung cancer cells via miRNA-424-3p downregulation, subsequent induction of PTEN signaling, and a significant decrease in PI3K/Akt expression [317]. As miRNAs are considered as key players in cisplatin resistance [318], their modulation by anti-tumor compounds paves the way for sensitivity. Exposing lung cancer cells to lidocaine induces miRNA-21 downregulation to promote PTEN expression, leading to PI3K/Akt suppression and cisplatin sensitivity [319]. One of the interesting points is the anti-tumor activity of propofol as an anesthetic agent. This compound is exclusively applied in cancer therapy, and it is capable of regulating different molecular pathways in cancer therapy, particularly miRNAs [320,321,322]. In non-small cell lung cancer, propofol downregulates miRNA-21 expression to induce apoptosis in a time- and dose-dependent manner. Upon miRNA-21 inhibition, propofol increases PTEN expression, which mediates anti-tumor activity against lung cancer cells [323]. Although a few studies have evaluated miRNA/PTEN axis regulation by anti-tumor compounds, it seems that this pathway is a novel target for impairing lung cancer growth (Figure 5) [324].

## 5. Conclusions and Remarks

In the present review, a mechanistic discussion of miRNA and PTEN interaction in lung and breast cancers was provided. We investigated this interaction in lung and breast cancers as the most malignant thoracic tumors. The results were in line with each other. PTEN loss occurs in both lung and breast cancers, leading to their progression via the activation of PI3K/Akt signaling, and downstream targets including EMT, GSK-3b, HIF-1a, and so on. miRNAs are divided into two main categories, including tumor-suppressing miRNAs that promote PTEN expression and tumor-promoting miRNAs that reduce PTEN expression. Furthermore, the miRNA/PTEN axis can be regulated by upstream mediators and anti-tumor compounds. LncRNAs and circRNAs are the most well-known regulators of the miRNA/PTEN axis in lung and breast cancers. Anti-tumor compounds promote the expression of tumor-suppressing miRNAs in inducing PTEN expression and suppressing cancer malignancy.

It is noteworthy that the expression of miRNA and PTEN as well as their interaction and capability of being used as diagnostic and prognostic factors in lung and breast cancers have been investigated. With respect to the fact that PTEN loss occurs in cancer patients, and miRNAs regulating PTEN have been identified, they can be used as reliable biomarkers. Novel therapeutics can be developed for application in clinical studies and treatment of lung and breast cancer patients. However, we are still at the beginning point, and more studies are needed to evaluate the miRNA/PTEN axis in these malignant tumors.

## Figures and Tables

**Table 1:** MicroRNAs (miRNAs) inhibiting PTEN in promoting breast cancer progression.

miRNA	Signaling Network	Outcomes	Refs.
**miRNA** **-106b** **miRNA** **-93**	PTEN/PI3K/Akt	Increasing cancer cell proliferation and metastasis PTEN downregulation Inducing PI3K/Akt signaling	[139]
**miRNA** **-182-5p**	-	Silencing miRNA-182-5p impairs cancer malignancy via PTEN upregulation	[127]
**miRNA** **-221/222**	PTEN/Akt	Promoting colony formation capacity Inducing Akt signaling via PTEN inhibition	[121]
**miRNA** **-19a/b**	PTEN/Akt/p21	Inhibiting cell cycle arrest at G1/S phase Binding to 3′-UTR of PTEN in reducing its expression P21 inhibition Upregulating PCNA and cyclin D1	[131]
**miRNA** **-9** **miRNA** **-155**	-	Involvement of these exosomal miRNAs in metastasis of breast cancer cells via PTEN downregulation	[112]
**miRNA** **-10b**	PTEN/Akt	Maintaining self-renewal capacity of breast cancer cells Akt hyperactivation via PTEN downregulation	[126]
**miRNA** **-221/222**	PTEN/Akt/NF-kB/COX-2	Enhancing stem cell-like features of breast cancer cells Increasing colony formation capacity Promoting stemness via ALDH1 upregulation PTEN inhibition Activating Akt/NF-kB/COX-2 axis	[140]
**miRNA** **-181c**	-	Increasing cancer growth by binding to 3′-UTR of PTEN	[109]
**miRNA** **-425-5p**	-	Association with poor prognosis of breast cancer patients Dually promoting cancer cell proliferation and metastasis PTEN inhibition	[115]
**miRNA** **-30a**	PTEN/Akt	Downregulating PTEN expression Providing condition for Akt phosphorylation Promoting cancer cell survival and growth	[122]
**miRNA** **-21**	PTEN/Akt/ERK1/2	Silencing miRNA-21 disrupts cancer metastasis (EMT) via PTEN upregulation and subsequent inhibition of Akt/ERK1/2	[141]
**miRNA** **-19a-3p**	-	miRNA downregulation by cold atmospheric plasma leads to breast cancer suppression via PTEN upregulation	[142]

**Table 2:** miRNAs inhibiting PTEN expression in enhancing lung cancer progression.

miRNA	Signaling Network	Outcomes	Refs
**miRNA** **-93**	LKB1/PTEN/CDKN1A/PI3K/Akt	Upregulation of miRNA-93 in lung cancer cells Association with proliferation and metastasis of cancer cells Inhibiting LKB1/PTEN/p21 axis Inducing PI3K/Akt	[171]
**miRNA** **-21**	PTEN/EMT	Reverse relationship with PTEN Promoting metastasis via EMT induction	[172]
**miRNA** **-21**	PTEN/Akt/GSK-3b	Increasing cyclin D1 and cyclin E1 expressions Enhancing cancer cell proliferation Promoting metastasis via EMT induction Activating Akt/GSK-3b signaling via PTEN downregulation	[173]
**miRNA** **-21**	-	Enhancing cell proliferation and invasion Apoptosis inhibition PTEN inhibition	[174]
**miRNA** **-26a**	PTEN/Akt	Enhancing metastasis via PTEN downregulation and the subsequent induction of Akt signaling	[175]
**miRNA** **-21**	-	Binding to 3′-UTR of PTEN Reducing the mRNA level of PTEN Promoting growth and metastatic features	[176]
**miRNA** **-205**	PTEN/Akt/mTOR	PTEN inhibition Activating Akt/mTOR signaling Increasing malignancy of lung cancer cells	[177]
**miRNA** **-183-5p**	PTEN/Akt/p53	Exerting oncogenic role Promoting Akt phosphorylation via PTEN downregulation Activating p53	[178]

**Table 3:** miRNAs regulating PTEN signaling in breast cancer chemotherapy.

miRNA	Chemotherapeutic Agent	Impact on Chemotherapy	Remarks	Refs.
**miRNA** **-93**	Doxorubicin	Resistance	PTEN downregulation EMT induction Increasing cancer metastasis and malignancy	[209]
**miRNA** **-202-5p**	Doxorubicin	Resistance	Enhancing tumor volume and progression Downregulating PTEN and subsequent induction of PI3K/Akt signaling	[107]
**miRNA** **-222**	Adriamycin	Resistance	Activation of PI3K/Akt signaling via PTEN downregulation Association with poor prognosis	[203]
**miRNA** **-222**	Adriamycin	Resistance	PTEN downregulation Inducing Akt signaling P27 inhibition Triggering the resistance of cancer cells to apoptosis	[191]
**miRNA** **-222** **miRNA** **-29a**	Adriamycin Doxorubicin	Resistance	Overexpression in drug-resistant cancer cells Association with PTEN downregulation	[204]
**miRNA** **-520h**	Paclitaxel	Resistance	Binding to OTUD3 and reducing its expression PTEN inhibition Paving the way for Akt induction	[212]
**miRNA** **-221/222**	Cisplatin	Resistance	Downregulation of miRNA-221/222 enhances cisplatin sensitivity Activation of p53/PTEN signaling following miRNA inhibition	[211]

**Table 4:** miRNAs affecting the response of lung cancer cells to chemotherapy.

miRNA	Chemotherapeutic Agent	Effect on Chemotherapy	Remarks	Refs.
**miRNA** **-181**	Cisplatin	Sensitivity	PTEN upregulation Inhibition of PI3K/Akt/mTOR signaling Apoptosis induction Disrupting cancer metastasis	[249]
**miRNA** **-29b-3p**	Cisplatin	Sensitivity	Disrupting cell viability Reducing proliferation Inducing apoptosis via Bax upregulation Triggering PTEN signaling	[228]
**miRNA** **-23a**	Erlotinib	Resistance	Silencing miRNA-23a restores PTEN expression to suppress PI3K/Akt signaling, leading to erlotinib sensitivity	[229]
**miRNA** **-134/487b/655**	Gefitinib	Resistance	Inducing TGF-b1 signaling in reducing PTEN expression Enhancing cancer metastasis via EMT induction Providing chemoresistance	[246]
**miRNA** **-21**	Gefitinib	Resistance	Reverse relationship between miRNA-21 and PTEN Activation of Akt and ERK signaling pathways Association with poor prognosis	[232]
**miRNA** **-21**	Cisplatin	Resistance	Hypoxia induces exsoaomal transfer of miRNA-21 Exerting PTEN inhibition	[233]
**miRNA** **-92b**	Cisplatin	Resistance	Establishing cancer proliferation Reducing sensitivity to chemotherapy PTEN inhibition	[248]
**miRNA** **-1269b**	Cisplatin	Resistance	Enhancing cancer cell growth Apoptosis inhibition Inducing PI3K/Akt signaling via PTEN downregulation	[237]
**miRNA** **-21**	EGFR-TKI	Resistance	Negative association with PTEN expression Triggering PI3K/Akt signaling Reducing chemosensitivity	[234]

## Data Availability

Not applicable.

## Abbreviations

SCLC: small cell lung cancer; NSCLC, non-small cell lung cancer; miRNAs, microRNAs; TGF-b, transforming growth factor-beta; EMT, epithelial-to-mesenchymal transition; RERG, ras-related and estrogen-regulated growth inhibitor; SP1, specificity protein 1; MRP1, multidrug resistance protein 1; PTEN, phosphatase and tensin homolog; 3^/^-UTR, 3^/^-untranslated region; mRNA, messenger RNA; lncRNAs, long non-coding RNAs; circRNAs, circular RNAs; Akt, protein kinase B; PI3K, phosphatidylinositol 3-kinase; mTOR, mammalian target of rapamycin; PIP2, phosphatidylinositol-4,5-biphosphate; PIP3, phosphatidyilinositol-3,4,5-triphosphate; PH, pleckstrin homology; NF-kB, nuclear factor-kappaB; CSCs, cancer stem cells; CICs, cancer-initiating cells; BCSCs, breast cancer stem cells; PAEs, phthalates; PCNA, proliferating cell nuclear antigen; Skp2, S-phase kinase-associated protein 2; HK2, hexokinase 2; PKM2, pyruvate kinase isozyme M2; PD-L1, programmed death-ligand 1; EZH2, enhancer of zeste homolog 2; HDAC9, histone deacetylase 9; IKK, IkB kinase; COX-2, cyclooxygenase-2; LC3, light chain-3; ATG5, autophagy-related gene 5; ceRNA, competing endogenous RNA; TRAIL, tumor necrosis factor-related apoptosis-inducing signal; CAFs, cancer associated fibroblasts; GAS5, growth arrest-specific transcript 5; CTD, cantharidin; MMP9, matrix metalloproteinase-9; CASC9, cancer susceptibility candidate 9; ERK, extracellular-signal regulated kinase; MMPs, matrix metalloproteinases; TNF, tumor necrosis factor; TNM, tumor node metastasis; XIST, X-inactive specific transcript.
